# Phthalates and asthma in children and adults: US NHANES 2007–2012

**DOI:** 10.1007/s11356-019-06003-2

**Published:** 2019-07-31

**Authors:** Chinonso Christian Odebeatu, Timothy Taylor, Lora E. Fleming, Nicholas J. Osborne

**Affiliations:** 1 European Centre for Environment and Human Health, Knowledge Spa, Royal Cornwall Hospital, University of Exeter Medical School, Truro, Cornwall, TR1 3HD UK; 2 School of Public Health and Community Medicine, University of New South Wales, Kensington, Sydney, 2052 Australia; 3 School of Public Health, The University of Queensland, Herston, Queensland 4006 Australia

**Keywords:** Phthalate metabolites, Mono-benzyl phthalate, Childhood asthma, Adult asthma, NHANES

## Abstract

Environmental exposure to phthalates may contribute to an increased risk of asthma in children and adults. We aimed to assess the direction and strength of the association between urinary phthalates metabolites and current asthma in children and adults that participated in the National Health and Nutrition Examination Survey (NHANES) 2007–2012. Data on ten urinary phthalate metabolites, self-reported questionnaires, spirometry measures, and covariates were obtained from 7765 participants (28.1% were children aged 6–17 years) taking part in the NHANES 2007–2012. Asthma was assessed using self-reported questionnaires for children and adults, and via spirometry measures for adults alone. We used crude and adjusted logistic regression models to estimate the odds ratios (ORs) and 95% confidence interval (CI) per one log_10_ unit change in the concentration of phthalate metabolites. We further modeled the effect modification by sex. Out of 10 metabolites, only mono-benzyl phthalate (MBzP) was positively associated with the prevalence of self-reported asthma in children, after adjusting for a range of potential confounders (odds ratio 1.54; 95% confidence interval 1.05–2.27). No significant relationship was observed for adults. The association of mono-ethyl phthalate (MEP) was modified by sex, with significantly increased odds of asthma among males [boys (2.00; 1.14–3.51); adult males (1.32; 1.04–1.69)]. While no other phthalates showed a positive relationship with current asthma in males, mono-(carboxynonyl) phthalate (MCNP) and mono-(3-carboxylpropyl) phthalate (MCPP) were inversely associated with spirometrically defined asthma in adult females. A sex-specific relationship in adults was evident when spirometry, but not self-reported measures were used to define asthma. We found no clear association between exposure to phthalates and current asthma, except for a significant relationship between MBzP metabolites and self-reported asthma in children. As a result, exposure to phthalates and asthma development and/or exacerbations remains controversial, suggesting a need for a well-designed longitudinal study.

**Electronic supplementary material:**

The online version of this article (10.1007/s11356-019-06003-2) contains supplementary material, which is available to authorized users.

## Introduction

For the past two decades, the prevalence of asthma has substantially increased in both the developed and developing countries (Osborne et al. 2017). The International Study of Asthma and Allergies in Childhood (ISAAC) study demonstrated that across 37 countries including the US and the UK, the average prevalence of asthma in 2006 amongst children aged 6–7 years was 12.6% (Asher et al. 2006). The Global Burden of Disease attributed to asthma is predicted to be about 11 million years of life lost (YLLs) and 25 million disability-adjusted life years (DALYs) per year (Osborne et al. 2017).

Asthma is a common chronic disease in children and adults characterised by airway inflammation and increased mucus production—leading to airway obstruction (Khalili et al. 2018). It is estimated by the US Centers for Disease Control and Prevention (CDC) that 6.8% of US working adults have current asthma—defined as having had at least one asthma attack or visit the emergency department (ED) for asthma in the past 12 months (Mazurek and Syamlal 2018). The disease is a potential threat to children’s growth and development including their educational achievement (Nurmagambetov et al. 2018). In 2017, the prevalence of asthma was reported in approximately 6.2 million children in the US—about 8.4% of children under the age of 18 (CDC 2017). The US annual economic costs associated with asthma have been estimated at $81 billion for 2013, including treatment costs and mortality costs valued using the value of statistical life and lost work and school days (Nurmagambetov et al. 2018). The root causes of asthma have not been fully elucidated, but genetic predisposition, and environmental factors including allergens and chemicals (such as phthalates) as well as gene-environment interactions, have been suggested as important risk factors for asthma pathogenesis and exacerbations (Wang et al. 2015; Sordillo et al. 2015; Surdu et al. 2006).

Phthalates are synthetic chemicals produced by reacting phthalic anhydride with different chain lengths of alcohol(s) which may vary from single chain alcohol (such as methanol) to multiple chain alcohol (such as tridecyl alcohol) (Benjamin et al. 2017). They are mainly classified into two types—high (HMW) and low molecular weight (LMW) phthalates—and their uses may in part depend on their molecular weight (Table 1) (Braun et al. 2013; Benjamin et al. 2017). Phthalates are omnipresent and are not covalently bound to the consumer products; they easily leach out and make their way to the environment (Tsai et al. 2012). Humans are exposed to these chemicals through several routes of exposure including water, breathing air, dermal contact, during medical treatment and, importantly, via food (Benjamin et al. 2017).

**Table 1:** Commonly used phthalates, their molecular weights and primary metabolites. Adapted from Braun et al. (2013) and Benjamin et al. (2017)

Commonly used phthalates (parent compound)	Abbreviation	Metabolites measured in epidemiological studies	Molecular weight (MW)	Uses/applications
High molecular weight (HMW) phthalates				
Di- (2-ethylhexyl) phthalate	DEHP	a) Mono-(2-ethylhexyl) phthalate (MEHP) b) Mono-(2-ethyl-5-oxohexyl) phthalate (MEOHP) c) Mono-(2-ethyl-5-hydroxylhexyl) phthalate (MEHHP) d) Mono-(2-ethyl-5-carboxylpentyl) phthalate (MECPP)	390.56	Plasticiser for PVC including medical tubing (blood bags, syringes and dialysis equipment), construction and automotive, some food packaging, flooring and floor tiles, toys, solvents in lip sticks, plastic films, gloves, shower curtains, wall covering, ethyl cellulose resin (such as electric wire, imitation leather, mould plastic products and rain wears.
Di-isononyl phthalate	DiNP	a) Mono-(carboxyloctyl) phthalate (MCOP) b) Mono-isononyl phthalate (MiNP)	418.61	PVC sheeting, building and construction materials, foot wears, sealing, several categories of toys (plastic books, ball, doll and cartoon characters), paints, automotive parts adhesives, printing ink for t-shirts, soap packaging, resins, and electrical wires and cables, etc.
Di-isodecyl phthalate	DiDP	Mono-(carboxylnonyl) phthalate (MCNP)	446.66	Plasticiser in PVC, pharmaceutical pills, food wrappers, plastic paste for coating, textile inks, PVC
				Flooring materials, hollow plastic products such as toys, exercise balls, and hoppers, and adhesives.
Di-n-octyl phthalate	DnOP	Mono (3-carboxylpropyl) phthalate (MCPP)	390.56	Plasticiser in PVC, paints, lacquers, adhesives, flooring tiles.
Benzylbutyl phthalate	BBzP	a) Mono-benzyl phthalate (MBzP) b) Mono (3-carboxylpropyl) phthalate (MCPP)	278.34	Cellulose, varnishes, toys, childcare articles, school supplies, children clothes, acetate plastics, personal care products (including nail polish and cosmetics) and fragrance ingredients.
Low molecular weight (LMW) phthalates				
Di-butyl phthalate	DBP	a) Mono-n-butyl phthalate (MnBP) b) Mono (3-carboxylpropyl) phthalate (MCPP)	278.34	Cellulose acetate plastics, personal care products, varnishes, pharmaceutical coatings and fragrance ingredients.
D-imethyl phthalate	DMP	Mono-methyl phthalate (MMP)	194.18	Fragrance ingredients for cosmetics, domestic and personal care product, adhesives, children’s toys, lacquers, paints, plastics and rubbers.
Di-ethyl phthalate	DEP	Mono-ethyl phthalate (MEP)	222.24	Personal care items (fragrances), pharmaceutical coatings and packaging, dyes, nail polish, perfumes as a solvent, ingredient in aspirin coating, surface lubricants in food, automotive parts, adhesives and plasticisers.

Although phthalates are easily bio-transformed and excreted (leading to lesser bioaccumulation), regular exposure in humans may exacerbate the risk of developing asthma or prolong its prevalence by binding with and activating peroxisome proliferator-activated receptors (PPARs) which mediate anti-inflammatory effects in the lungs and immune systems (Bølling et al. 2013); increasing the proliferation of the bronchial muscle cells which may lead to airways remodelling (Kuo et al. 2011); promoting the production of pro-inflammatory cytokines IL-6 and IL-8 in the airway epithelial cells (Jepsen et al. 2004); and/or; acting as an adjuvants by enhancing macrophage production of inflammatory cytokines and chemokines (Nishioka et al. 2012).

Several epidemiological studies have demonstrated that regular exposure to phthalates is associated with an increased risk of non-communicable chronic diseases including cardiovascular diseases and diabetes (Dong et al. 2017; Bai et al. 2017). Limited information is known about the association between phthalates exposure and the prevalence of asthma (Benjamin et al. 2017), with available evidence producing inconsistent results. A meta-analysis demonstrated that post-natal exposure to di-(2-ethylhexyl) phthalate (DEHP) and butylbenzyl phthalate (BBzP) from dust and prenatal urinary mono-benzyl phthalate (MBzP) were significantly associated with childhood asthma (Li et al. 2017).

In contrast, a recent study has shown that both LMW and HMW phthalates (including DEHP) were not associated with the report of doctor-diagnosed asthma (Vernet et al. 2017). Previous research found that the urinary concentration of MBzP metabolite was associated with self-reported asthma in adults but not in children (Hoppin et al. 2013). These inconsistencies need to be addressed with more research into the potential association between phthalates and asthma in children and adults using a large cross-sectional secondary data and better outcome measures (for example spirometry and questionnaire data).

In addition, the development and/or exacerbation of asthma may be sex-specific. Whilst the prevalence of asthma, in general, is greater in females than in males (CDC 2008), investigation at a specific time point revealed otherwise. Before age 13–14 years, the incidence and prevalence of asthma with increased wheeze, use of asthma medications and serum IgE level are greater among boys than among girls (Wijga et al. 2011; Almqvist et al. 2008; Bjornson and Mitchell 2000). By contrast, studies through puberty and beyond have found a greater increase in the incidence and prevalence of asthma among adolescent and young adult females (CDC 2008; De Marco et al. 2000). Importantly, a prospective cohort study demonstrated that the relationship between phthalates and asthma may be modified by sex (Buckley et al. 2018), with 5-year-old boys at increased odds of asthma occurrence following exposures to mono-(2-ethylhexyl) phthalate (MEHP) and mono-ethyl phthalate (MEP) (Ku et al. 2015).

In the current study, we polled the National Health and Nutrition Examination Survey (NHANES) 2007–2012 data to examine the direction and strength of the association between urinary phthalate metabolites and current asthma in children and adults. As a secondary aim, we stratified the data based on the participant sex, to investigate whether the effect measure was modified by sex in both children and adults.

## Methods

### Study population

NHANES is a nationally representative, multi-stage, population-based, cross-sectional study carried out by the US National Centre for Health Statistics (NCHS). It was designed to assess the health and nutritional status of civilian, non-institutionalised children and adults in the US. Respondents for our study were children aged between 6 and 17 years, and adults aged 18 and 79 years, who were randomly selected by the NHANES for urinary phthalates measurement; and who had complete information on self-reported questionnaires, spirometry and confounding variables. Participants aged 80 years and over were excluded in order to reduce biases resulting from the non-representation of the older adults who are institutionalised after 80 years.

Data were pooled from three independent cross-sectional waves (2007–2008, 2009–2010, and 2011–2012), providing an initial total sample of 30,442 participants (11,823 children and 18,619 adults). Urinary phthalate concentrations were determined for 7765 subsets (2180 children and 5585 adults); therefore, only these participants were used for analysis. All selected participants provided informed consent in writing during the period of recruitment (NHANES 2017).

### Measurement of phthalate metabolites

Phthalate metabolites were measured in a spot urine sample of a randomly selected one third sub-sample of the study respondents. These collected samples were frozen at the temperature of − 20 °C and shipped to the division of Environmental Health Laboratory Sciences, National Centre for Environmental Health, CDC for the analysis of various phthalate metabolites. In order to reduce the possibility of exposure misclassification (James-Todd et al. 2016), phthalate metabolites were measured instead of their parent compound. A full description of the analytical methods employed for the measurement of phthalates metabolites have been described elsewhere (Laboratory Procedure Manual 2013).

A combination of phthalate metabolites that have been previously studied and those that were measured in all the three cross-sectional waves, with more than 60% of the sample concentrations at or above the limit of detection (LOD) (James-Todd et al. 2016) were selected for this project. These included ten (10) phthalate metabolites: mono-(carboxynonyl) phthalate (MCNP), mono-(2-ethyl-5-carboxypentyl) phthalate (MECPP), MEHP, mono-(2-ethyl-5-hydroxylhexyl) phthalate (MEHHP), mono-(2-ethyl-5-oxohexyl) phthalate (MEOHP), mono-n-butyl phthalate (MnBP), mono-iso-butyl phthalate (MiBP), MBzP, MEP and mono-(3-carboxylpropyl) phthalate (MCPP).

Given that the LOD for phthalate metabolites differed across each survey cycle, the maximum limit of detection (LOD_max_) was used to standardise each phthalate detection limits in the three cross-sectional waves (Varshavsky et al. 2018). Thus, all concentrations below the LOD_max_ were substituted with the value of LOD_max_ divided by the square root of two (Varshavsky et al. 2018).

### Asthma data

#### Self-reported questionnaire data

NHANES collected information on asthma and associated symptoms using a self-administered questionnaire completed at the NHANES clinic visit. Following the recommendation from the European birth cohort study (Carlsen et al. 2012), current asthma was defined by respondents giving a positive response to both questions: “Has a doctor or other health professional ever told you that you have asthma?” and “In the past 12 months (have you/has SP) had wheezing or whistling in (your/his/her) chest?”

#### Spirometry data

Spirometry data were available in all cross-sectional waves and were also used for asthma determination. Participants aged 6 to 79 years were considered eligible for spirometry testing. Respondents were excluded if they: had current chest pain or physical problems with forceful expiration; had recent chest, eye or abdominal surgery; had a heart problem (such as heart attack), stroke or tuberculosis; were taking supplementary oxygen; had a collapsed lung or detached retina; had painful ear problems or had coughed up blood recently (NHANES 2014).

Spirometry testing for eligible participants was performed following the procedures recommended by the American Thoracic Society (ATS). The protocol and procedures for spirometry testing have been described elsewhere (NHANES 2008). The baseline spirometry results of forced expiratory volume in 1 s (FEV_1_), forced vital capacity (FVC) and FEV_1_/FVC% were determined by adopting a normal equation for spirometry parameters of the US population which takes into account each respondent’s age, sex, weight, height and race/ethnicity (NHANES 2014).

Based on the guidelines set from the International Consensus Statement between the ATS and the European Respiratory Society (ERS) which suggest the presence of airflow obstruction when the FEV1/FVC ratio was less than 70% (Cerveri et al. 2008), current untreated asthma was defined via spirometry results as respondents with an FEV1/FVC of < 70% (Abo-Zaid et al. 2018). Analyses using self-reported but not with spirometry data were performed for children. This is because accuracy and precision suffer in spirometry testing involving children (Murray et al. 2016).

#### Confounding variables

Information on covariates was obtained from the NHANES. These covariates were determined using the self-reported questionnaire, physical examination, and laboratory measurements. Age, sex, race/ethnicity and poverty status (which serves as a proxy for socioeconomic status (SES)) were ascertained via questionnaire. Poverty status was defined by the poverty income ratio (PIR) which was calculated by dividing the family income by the poverty guidelines of a specific survey year. Race/ethnicity was classified as “non-Hispanic white” (referent group), “non-Hispanic black”, “Mexican-American,” and “Other”.

Waist circumference in centimetres (cm) was used as a measure of overweight since it gives a better measure of obesity-related health risks than body mass index (BMI) (Janssen et al. 2004). Urinary creatinine concentrations were measured using Roche/Hitachi Modular P chemistry analyser and Synchron CX3 clinical analyser (Beckman, CA, USA). Serum cotinine level (a biomarker for smoking status) was categorised as < LOD (< 0.015) nanogram/ml (ng/ml) (referent), low levels (≥ 0.015–10 ng/ml) and high levels (≥ 10 ng/ml).

#### Statistical analysis

The Spearman’s rank correlation coefficient was used to examine phthalate metabolite correlation. Phthalate metabolite was considered to have a strong correlation with a Spearman’s correlation coefficient (*r*_*s*_) greater than or equal to 0.7 (*r*_*s*_ ≥ 0.7). MEHP (a primary metabolite) and MECPP, MEHHP and MEOHP (secondary metabolites) of DEHP were strongly correlated with one another (with a value of *r*_*s*_ between 0.73–0.98) (Additional file 1: Table S1). Therefore, these metabolites were not separately analysed given their strong correlation and common source; the molar sum of DEHP denoted as “ΣDEHP”, was used instead (Hoppin et al. 2013).

Sampling weights, stratification and clustering provided in the NHANES study were applied to all statistical analysis in order to account for the complex, multistage sampling design employed in the selection of the representative non-institutionalised US population as well as obtaining accurate estimates that will not overstate the statistical significance. Following the NHANES analytical guidelines (Johnson et al. 2013), a new sampling weight for the combined survey cycle was constructed by dividing the 2-year weights for each cycle by 3 which was applied to the data via the Stata command [svyset] prior to analysis.

Descriptive statistics (weighted means, standard deviation, weighted percentages and 95% confidence interval (CI)) were used to describe the demographics of all children and adults and their respective subsets with measured urinary phthalate metabolite concentrations. The distribution of urinary phthalate metabolites were presented for both children and adults using weighted geometric means, 95% CI and percentiles.

Logistic regression models (models 1 and 2) were used to determine the cross-sectional measure of the association between urinary phthalate metabolites (continuous) and current asthma (dichotomous outcome) by estimating the odds ratios (ORs) and 95% CIs per one log_10_ unit change in the concentration of phthalate metabolites. Model 1 was presented as unadjusted ORs and 95% CI. Model 2 was adjusted for urinary creatinine (log_10_ transformed, continuous) in addition to other potential confounding variables.

Potential confounders included in this analysis were those suggested as being linked with phthalate metabolites and/or asthma (Hoppin et al. 2013; James-Todd et al. 2016; Gascon et al. 2015; Buckley et al. 2018). These variables included age, sex, race/ethnicity, waist circumference, PIR, cotinine and urinary creatinine. The analysis was further stratified by sex for both children and adults by applying similar statistical modeling.

In order to assess the robustness of our findings, a sensitivity analysis was performed. Exposure-response relationships were examined by modeling the associations between tertiles of phthalate creatinine-corrected concentrations and asthma, with the lowest tertile considered as the reference category (Buckley et al. 2018). Tertiles were categorised separately for children and adults such that each tertile contained an equal number of participants. While results for each phthalate metabolite were presented as crude and adjusted ORs and 95% CIs, only adjusted models were shown for effect modifications by sex and sensitivity analyses. All statistical analyses were conducted using STATA version 15.0 (College Station, TX, USA).

## Results

The demographic characteristics of all children (*n* = 11,823) and the subset with measured urinary phthalate concentrations (*n* = 2180), who participated in the NHANES 2007–2012 are shown in Table 2. Approximately 8% of children in both groups had self-reported asthma. With spirometry measures, the proportion of respondents with current asthma dropped to less than 2%. The weighted proportions of all children belonging to any race or living below the poverty threshold [poverty-to-income ratio (PIR)] were somewhat similar to those with measured phthalate metabolites.

**Table 2:** Demographic and asthma status for all children and subsets sampled for phthalate concentrations, NHANES 2007–2012

Children aged 6 to < 18 years		
Characteristics	All participants (*n* = 11823)	Participants sampled for phthalate concentrations (*n* = 2180)
Age at screening (years), weighted mean (SD, 95% CI)	8.6 (5.2, 8.44–8.74)	11.5 (3.4, 11.27–11.71)
Sex		
Male, weighted % (*n*, 95% CI)	50.8 (6037, 49.26–52.25)	50.1 (1095, 47.09–53.00)
Female, weighted % (*n*, 95% CI)	49.2 (5786, 47.75–50.74)	49.9 (1085, 46.99–52.91)
Race/ethnicity, weighted % (*n*, 95% CI)		
Non-Hispanic whites	55.5 (3646, 50.57–60.39)	56.1 (619, 50.38–61.62)
Non-Hispanic Blacks	14.3 (2801, 11.99–17.04)	14.7 (551, 12.13–17.73)
Mexican American Hispanic	15.0 (2983, 12.04–18.63)	14.8 (537, 11.48–18.77)
Others	15.1 (2575, 12.90–17.58)	14.4 (473, 11.74–17.66)
Waist circumference (cm), weighted mean (SD, 95% CI)	67.8 (16.2, 67.25–68.34)	73.1 (15.4, 72.10–73.99)
Family income-to-poverty ratio (PIR), weighted % (n, 95% CI)		
Below poverty (PIR < 1), weighted % (*n*, 95% CI)	24.4 (3823, 21.97–27.03)	23.7 (668, 20.85–26.82)
At or above poverty (PIR ≥ 1),	75.6 (7052, 72.97–78.03)	76.3 (1333, 73.18–79.15)
Urinary creatinine (mg/dL), weighted mean (SD, 95% CI)	119.2 (74.2, 114.76–123.69)	118.6 (75.3, 113.76–123.36)
Cotinine levels (ng/mL), weighted % (*n*, 95% CI)		
< LOD (< 0.015)	25.9 (1554, 23.03–29.13)	26.8 (457, 22.94–30.99)
Low (≥ 0.015 to < 10)	70.4 (4855, 67.15–73.42)	68.7 (1322, 64.72–72.47)
High (≥ 10)	3.7 (184, 2.96–4.50)	4.5 (63, 3.18–6.33)
Current asthma based on self-reported questionnaire, weighted % (*n*, 95% CI)		
Yes	7.6 (800, 7.13–8.14)	8.1 (177, 6.80–9.71)
No	92.4 (9700, 91.86–92.87)	91.9 (2003, 90.29–93.20)
Current asthma based on spirometry FEV_1_/FVC cutoff, weighted % (*n*, 95% CI)		
Yes	1.7 (110, 1.28–2.12)	1.5 (34, 1.02–2.07)
No	98.3 (5737, 97.88–98.72)	98.6 (1877, 97.93–98.98)

*SD* standard deviation, *CI* confidence interval, *LOD* limit of detection, *FEV1* forced expiratory volume in 1 s, *FVC* forced vital capacity

Of the 30,442 respondents in the NHANES 2007–2012 cross-sections, a total of 61.2% (*n* = 18,619) were adults - with approximately a third (*n* = 5585) of the participants sub-sampled for phthalate metabolite levels (Table 3). Although adults with detectable phthalate metabolite values were slightly younger when compared with all adults, there was no difference in the weighted proportions of the subjects belonging to any race or living below the poverty threshold [poverty-to-income ratio (PIR) < 1).

**Table 3:** Demographic and asthma status for all adults and subsets sampled for phthalate concentrations, NHANES 2007–2012

Adults aged 18 to < 80 years		
Characteristics	All participants (*n* = 18619)	Participants sampled for phthalate concentrations (n = 5585)
Age at screening (years), weighted mean (SD, 95% CI)	46.1 (17.3, 45.33–46.82)	44.2 (16.1, 43.36–45.00)
Sex		
Male, weighted % (*n*, 95% CI)	48.3 (9140, 47.59–49.06)	49.4 (2804, 47.95–50.94)
Female, weighted % (*n*, 95% CI)	51.7 (9479, 50.94–52.41)	50.6 (2781, 43.37–52.05)
Race/ethnicity, weighted % (*n*, 95% CI)		
Non-Hispanic whites	67.6 (8044, 63.20–71.62)	67.1 (2323, 62.74–71.25)
Non-Hispanic Blacks	11.6 (4050, 9.60–13.84)	11.9 (1271, 9.80–14.33)
Mexican American Hispanic	8.4 (2913, 6.48–10.75)	8.3 (893, 6.36–10.78)
Others	12.5 (3612, 10.70–14.61)	12.7 (1098, 10.81–14.81)
Waist circumference (cm), weighted mean (SD, 95% CI)	97.9 (16.2, 97.27–98.43)	97.8 (16.7, 97.05–98.60)
Family income-to-poverty ratio (PIR), weighted % (*n*, 95% CI)		
Below poverty (PIR < 1),	16.1 (4003, 14.56–17.78)	16.2 (1233, 14.20–18.50)
At or above poverty (PIR ≥ 1),	83.9 (12834, 82.15–85.44)	83.8 (3837, 81.50–85.80)
Urinary creatinine (mg/dL), weighted mean (SD, 95% CI)	120.8 (77.9, 117.84–123.81)	121.4 (78.7, 118.28–124.49)
Cotinine levels (ng/mL), weighted % (*n*, 95% CI)		
< LOD (< 0.015)	24.9 (3724, 23.28–26.69)	25.1 (1130, 22.90–27.35)
Low (≥ 0.015 to < 10)	50.0 (8844, 48.47–51.50)	49.2 (2705, 46.83–51.49)
High (≥ 10)	25.1 (4216, 23.58–26.60)	25.8 (1379, 23.60–28.10)
Current asthma based on self-reported questionnaire, weighted % (*n*, 95% CI)		
Yes	5.9 (1123, 5.29–6.75)	5.7 (349, 4.93–6.47)
No	94.0 (17,463, 93.24–94.71)	94.3 (5227, 93.53–95.07)
Current asthma based on spirometry FEV_1_/FVC cut-off, weighted % (n, 95% CI)		
Yes	13.4 (1818, 12.42–14.52)	13.6 (610, 12.19–15.05)
No	86.6 (12,353, 85.48–87.58)	86.4 (4966, 84.95–87.81)

*SD* standard deviation, *CI* confidence interval, *LOD* limit of detection, *n* number of observation, *FEV*_*1*_ forced expiratory volume in 1 s, *FVC* forced vital capacity

Self-reported asthma was seen in nearly 6% of both groups. With the spirometry measure, the proportion of asthmatics was more than doubled. This is expected given the nature of the US healthcare system with many adults in the US without health insurance and thus with undiagnosed asthma (Baldacci et al. 2015).

All participants (children and adults) had detectable concentrations of both LMW and HMW phthalate metabolites (detection frequency > 60%) (Tables 4 and 5). With regard to LMW phthalates, MEP had the highest mean concentrations for both children and adults. For HMW phthalates, MECPP showed the highest in both groups.

**Table 4:** Distribution of urinary phthalate concentrations for children (aged 6 to < 18 years), NHANES 2007–2012

Percentile											
Metabolite (ng/mL)	Sample size, *N*	LOD_max_ (ng/mL)	≥ LOD_max_ (%)^a^	Weighted geometric mean (95% CI)	Min	5th	25th	50th	75th	95th	Max
LMW phthalate											
MEP	2106	0.6	99.9	45.2 (39.85–50.45)	< LOD_max_	6.9	22.8	49.5	129.1	588.9	7633.2
MiBP	2106	0.3	99.6	9.7 (9.05–10.37)	< LOD_max_	1.5	5.4	11.5	22.2	56.2	1163.3
MnBP	2106	0.6	98.4	18.2 (16.56–19.89)	< LOD_max_	2.3	10.4	22.2	44.4	118.3	101013
HMW phthalate											
MBzP	2106	0.3	99.4	10.6 (9.55–11.67)	< LOD_max_	1.2	5.0	12.3	27.3	86.1	617.18
MCNP	2106	0.5	96.3	3.0 (2.72–3.19)	< LOD_max_	0.5	1.7	3.1	5.5	14.6	334
MCPP	2106	0.2	98.5	4.0 (3.56–4.39)	< LOD_max_	0.6	1.9	3.8	8.0	24.9	1425.8
ΣDEHP^b^	2106	–	–	0.2 (0.17–0.21)	0.004	0.03	0.10	0.20	0.40	1.43	15.57
MEHP	2106	1.1	66.7	2.1 (1.96–2.23)	< LOD_max_	< LOD_max_	< LOD_max_	1.9	4.2	15.3	204.7
MEHHP	2106	0.7	99.4	16.1 (14.58–17.65)	< LOD_max_	2.2	7.8	17.2	36.5	133.3	1672
MECPP	2106	0.5	100	27.1 (24.67–29.56)	< LOD_max_	5.0	14.6	28.4	56.4	194.1	1871
MEOHP	2106	0.6	99.1	10.2 (9.30–11.17)	< LOD_max_	1.5	5.1	11.2	22.8	76.8	1175.1

**Table 5:** Distribution of urinary phthalate concentrations for adults (aged 18 to < 80 years), NHANES 2007–2012

Percentile											
Metabolite (ng/mL)	Sample size, *N*	LOD_max_ (ng/mL)	≥ LOD_max_ (%)^a^	Weighted geometric mean (95% CI)	Min	5th	25th	50th	75th	95th	Max
LMW phthalate											
MEP	5417	0.6	99.9	63.6 (58.95–68.35)	< LOD_max_	6.8	26.1	72.1	224.3	1285	31,660
MiBP	5417	0.3	98.7	6.5 (6.12–6.90)	< LOD_max_	0.9	3.8	8.1	15.5	40.0	627
MnBP	5417	0.6	97.2	11.9 (10.96–12.79)	< LOD_max_	1.3	6.8	15.0	30.8	84.8	25,863
HMW phthalate											
MBzP	5417	0.3	97.8	5.3 (4.93–5.63)	< LOD_max_	0.6	2.5	5.9	13.4	42.3	450.2
MCNP	5417	0.5	92.7	2.6 (2.42–2.72)	< LOD_max_	< LOD	1.2	2.4	4.8	16.2	730.25
MCPP	5417	0.2	97.4	2.8 (2.54–2.99)	< LOD_max_	0.3	1.2	2.6	5.6	22.4	2597.3
ΣDEHP^b^	5417	–	–	0.1 (0.13–0.15)	0.003	0.02	0.07	0.14	0.30	1.1	106.72
MEHP	5417	1.1	62.8	2.1 (1.92–2.20)	< LOD_max_	< LOD_max_	< LOD_max_	1.7	4.0	16.9	1252.7
MEHHP	5417	0.7	98.5	12.5 (11.52–13.52)	< LOD_max_	1.6	5.9	12.8	27.3	116.3	9326.1
MECPP	5417	0.5	99.8	19.5 (17.99–20.92)	< LOD_max_	3.1	9.5	19.8	40.7	148.1	15828
MEOHP	5417	0.6	97.8	7.5 (6.90–8.07)	< LOD_max_	1.0	3.6	7.7	16.1	62.1	6079.9

*N* number of participants/urinary samples, *LOD* limit of detection, *Min* minimum, *5th* 5th percentile, *25th* 25th percentile, *50th* 50th percentile, *75th* 75th percentile, *95th* 95th percentile, *Max* maximum, *LMW* low molecular weight, *MMP* mono-n-methyl phthalate, *MEP* mono-ethyl phthalate, *MiBP* mono-isobutyl phthalate, *MnBP* mono-n-butyl phthalate, *HWM* high molecular weight, *MiNP* mono-isononyl phthalate, *MBzP* mono-benzyl phthalate, *MCNP* mono (carboxylnonyl) phthalate, *MCPP* mono-(3-carboxylpropyl) phthalate, *DEHP* di(2-ethylhexyl) phthalate, *MEHP* mono-(2-ethylhexyl) phthalate, *MEHHP* mono-(2-ethyl-5-hydroxylhexyl) phthalate, *MECPP* mono-(2-ethyl-5-carboxylpentyl) phthalate, *MEOHP* mono-(2-ethyl-5-oxohexyl) phthalate

^a^Percentage of phthalate metabolite concentrations at or above the maximum limit of detection (< LOD_max_). All concentrations below the LOD_max_ (< LOD_max_) were substituted with a value of LOD_max_ divided by square root of two (√2)

^b^ΣDEHP: molar sum of DEHP metabolites (MEHP, MEHHP, MECPP and MEOHP) expressed in μmol/L.

**Fig. 1:**
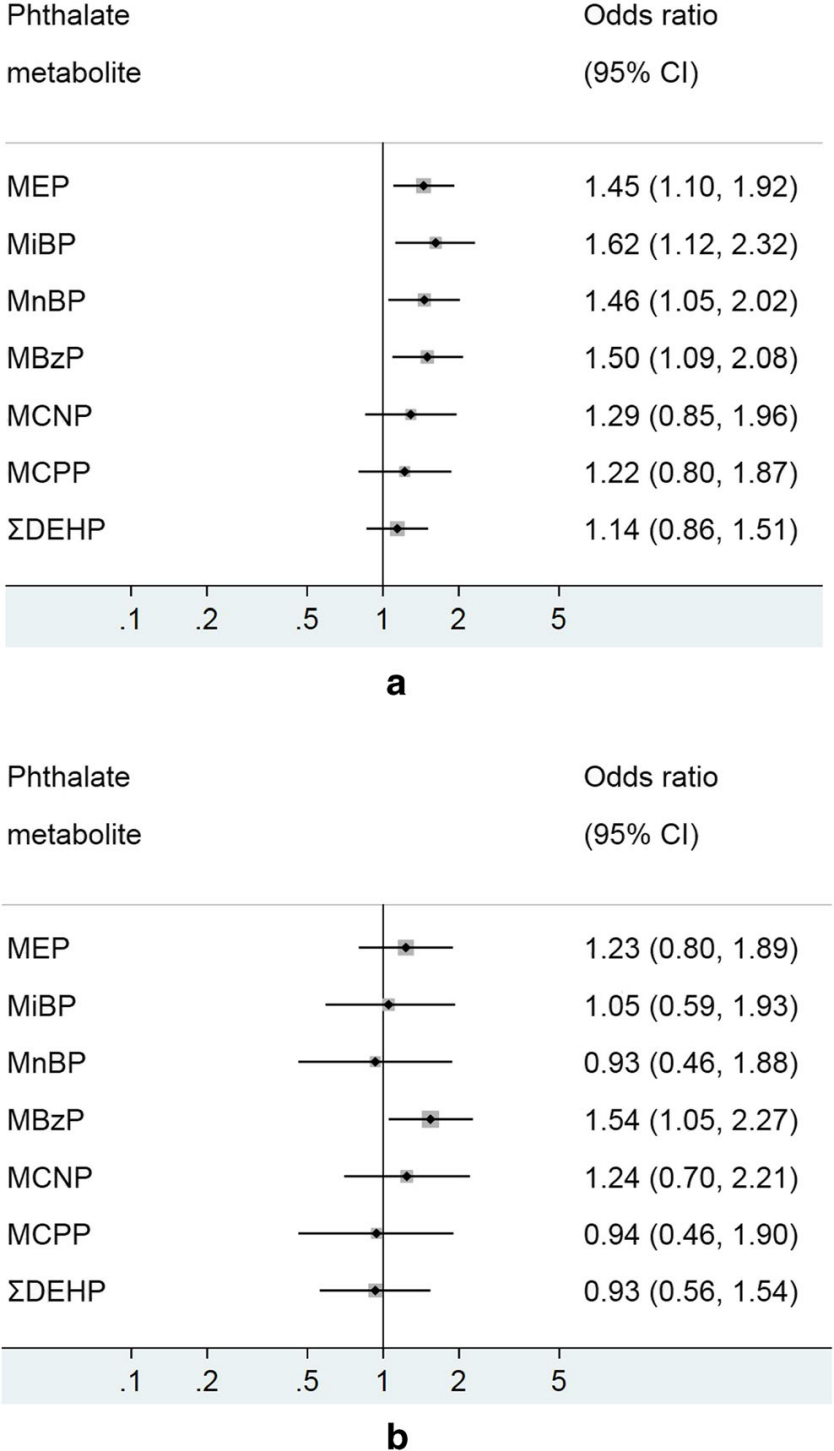
**a** Model 1 (crude) - associations of urinary phthalate metabolites with self-reported asthma in children, NHANES 2007–2012. Logistic regression modeling was used to access the effect of individual phthalate metabolites on asthma prevalence, with an odds ratio (OR) presented for 1 log_10_ unit change in urinary phthalate concentration. **b** Model 2 (adjusted)—associations of urinary phthalate metabolites with self-reported asthma in children, NHANES 2007–2012. Logistic regression modeling was used to access the effect of individual phthalate metabolites on asthma prevalence, with an OR presented for 1 log_10_ unit change in urinary phthalate concentration. MEP mono-ethyl phthalate, MiBP mono-isobutyl phthalate, MnBP mono-n-butyl phthalate, MBzP mono-benzyl phthalate, MCNP mono-(carboxynonyl) phthalate, MCPP mono-(3-carboxylpropyl) phthalate, ΣDEHP molar sum of DEHP metabolites (MEHP, MEHHP, MECPP and MEOHP). All models were adjusted for age, sex, ethnicity/race, waist circumference, cotinine, poverty and urinary creatinine

The crude and adjusted model of associations between the different phthalate metabolites and self-reported asthma in children are shown in Fig. 1 a and b, respectively. Self-reported childhood asthma was positively associated with MEP (1.45; 1.10–1.92), MiBP (1.62; 1.12–2.32), MnBP (1.46; 1.05–2.02) and MBzP (1.50; 1.09–2.08) in the crude analysis; with only MBzP (1.54; 1.05–2.27) reaching statistical significance after adjusting for confounding variables.

**Fig. 2:**
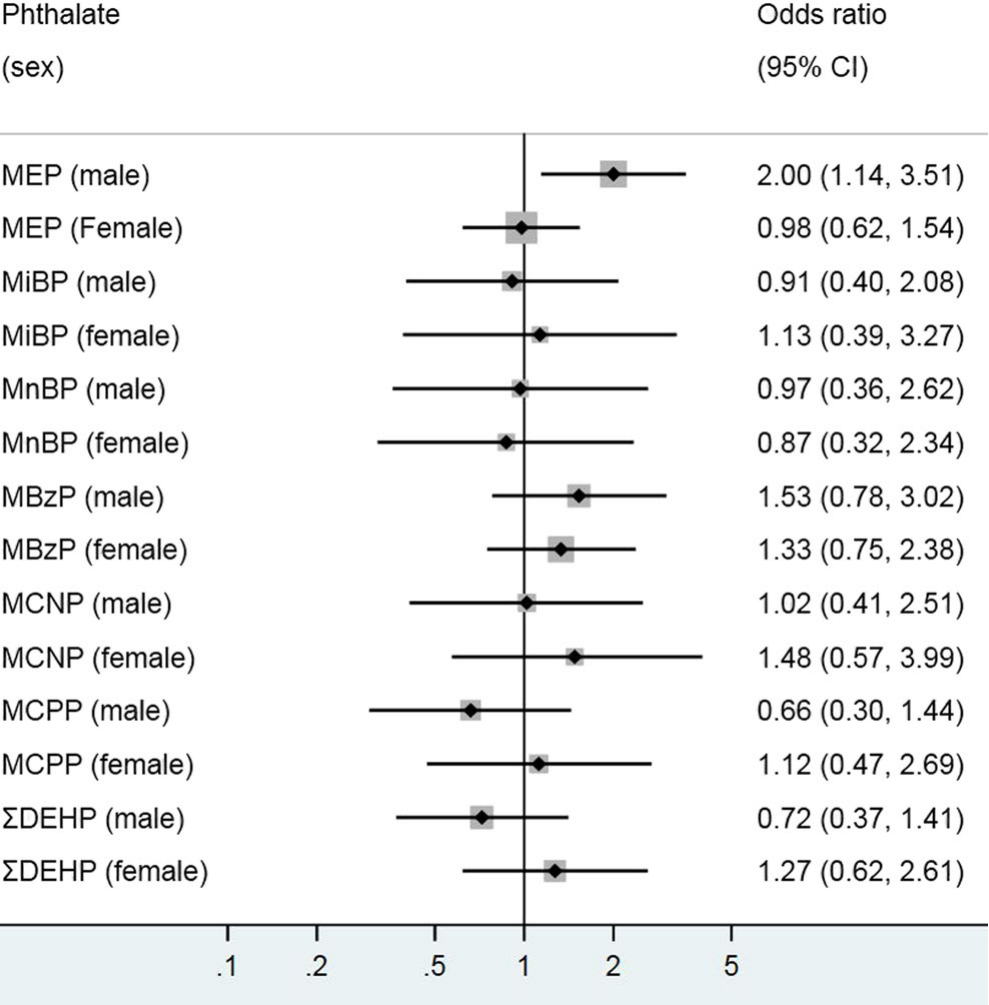
Associations between urinary phthalate metabolites and asthma (self-reported) in children stratified by sex. Logistic regression modeling was used to access the effect of individual phthalate metabolites on asthma prevalence, with odds ratio (OR) presented for 1 log_10_ unit change in urinary phthalate concentration. MEP mono-ethyl phthalate, MiBP mono-isobutyl phthalate, MnBP mono-n-butyl phthalate, MBzP mono-benzyl phthalate, MCNP mono-(carboxynonyl) phthalate, MCPP mono-(3-carboxylpropyl) phthalate, ΣDEHP molar sum of DEHP metabolites (MEHP, MEHHP, MECPP and MEOHP). All models were adjusted for age, race/ethnicity, waist circumference, poverty, urinary creatinine and cotinine

Stratification by child’s sex revealed that only the association between MEP and current asthma was modified, with a significant positive relationship among boys (2.00; 1.14–3.51), but not among girls (Fig. 2). Effect modification was not observed for MBzP, despite the significant relationship found in the overall model.

**Fig. 3:**
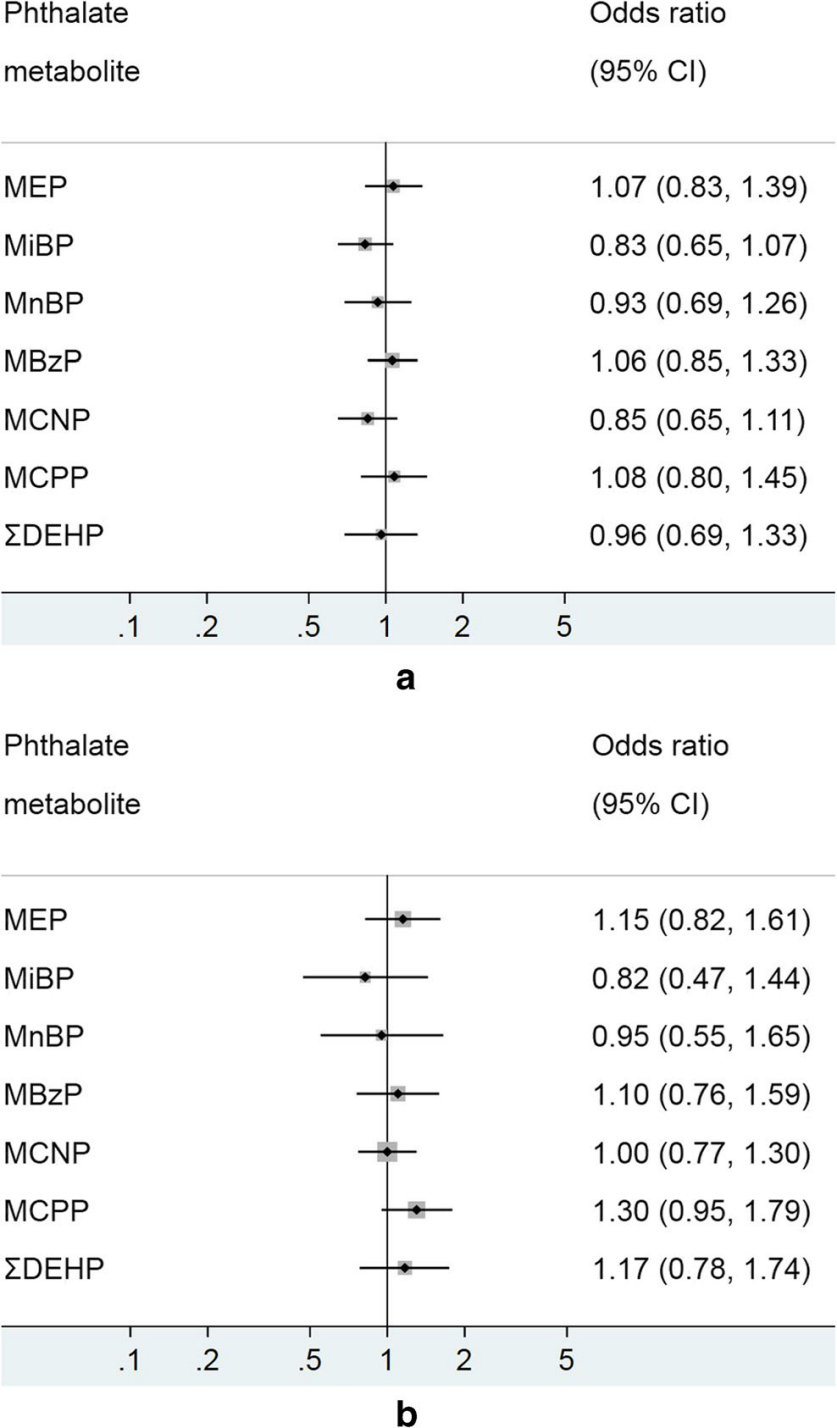
**a** Model 1 (crude)—associations of urinary phthalate metabolites with self-reported asthma in adults, NHANES 2007–2012. Logistic regression modeling was used to access the effect of individual phthalate metabolites on asthma prevalence, with odds ratio (OR) presented for 1 log_10_ unit change in urinary phthalate concentration. **b** Model 2 (adjusted) - associations of urinary phthalate metabolites with self-reported asthma in children, NHANES 2007–2012. Logistic regression modeling was used to access the effect of individual phthalate metabolites on asthma prevalence, with odds ratio (OR) presented for 1 log_10_ unit change in urinary phthalate concentration. MEP mono-ethyl phthalate, MiBP mono-isobutyl phthalate, MnBP mono-n-butyl phthalate, MBzP mono-benzyl phthalate, MCNP mono-(carboxynonyl) phthalate, MCPP mono-(3-carboxylpropyl) phthalate, ΣDEHP molar sum of DEHP metabolites (MEHP, MEHHP, MECPP and MEOHP). All models were adjusted for age, sex, ethnicity/race, waist circumference, cotinine, poverty and urinary creatinine

**Fig. 4:**
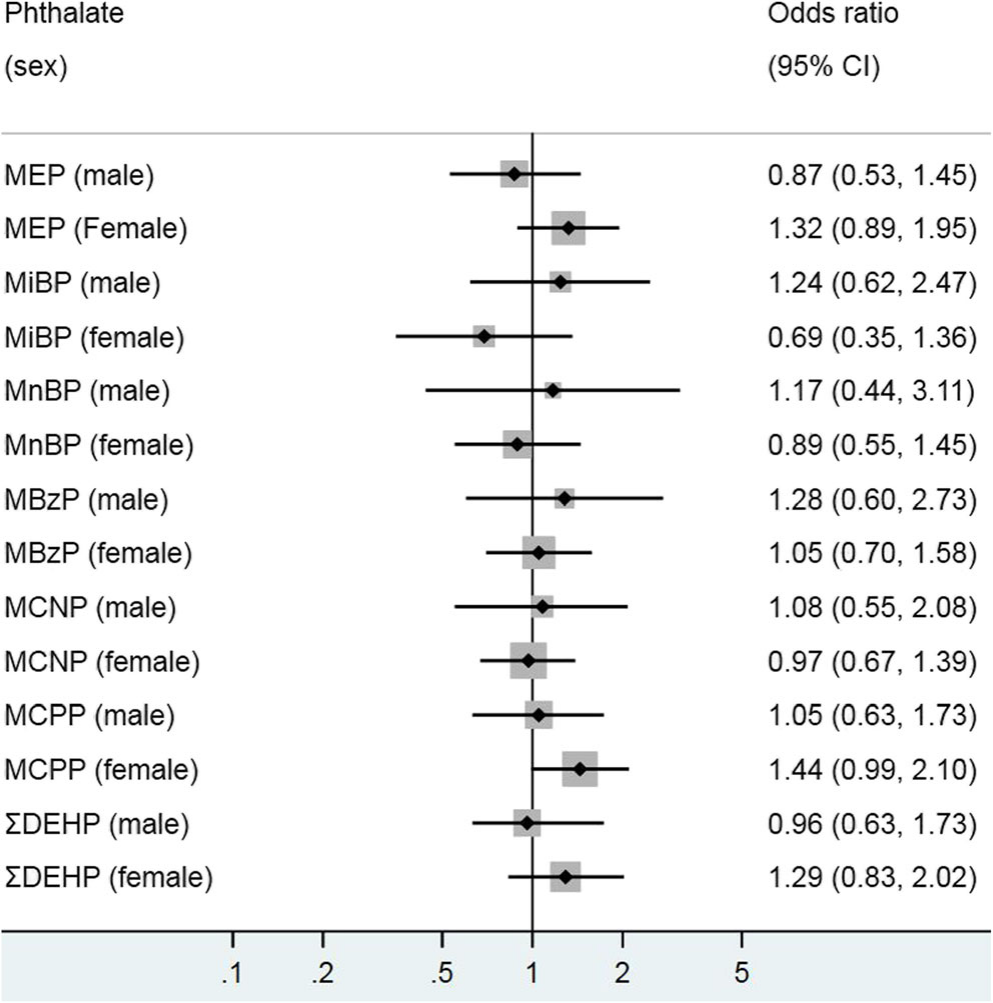
Associations between urinary phthalate metabolites and asthma (self-reported) in adults stratified by sex. Logistic regression modeling was used to access the effect of individual phthalate metabolites on asthma prevalence, with odds ratio (OR) presented for 1 log_10_ unit change in urinary phthalate concentration. MEP mono-ethyl phthalate, MiBP mono-isobutyl phthalate, MnBP mono-n-butyl phthalate, MBzP mono-benzyl phthalate, MCNP mono-(carboxynonyl) phthalate, MCPP mono-(3-carboxylpropyl) phthalate, ΣDEHP molar sum of DEHP metabolites (MEHP, MEHHP, MECPP and MEOHP). All models were adjusted for age, race/ethnicity, waist circumference, urinary creatinine and cotinine

The crude and adjusted analyses of associations between different phthalate metabolites and self-reported asthma in adults are shown in Fig. 3 a and b, respectively. No phthalate metabolite showed a clear significant association with self-reported asthma in either the crude or the adjusted models. Effect modification by adult sex was not observed between any phthalate metabolites and self-reported asthma (Fig. 4).

**Fig. 5:**
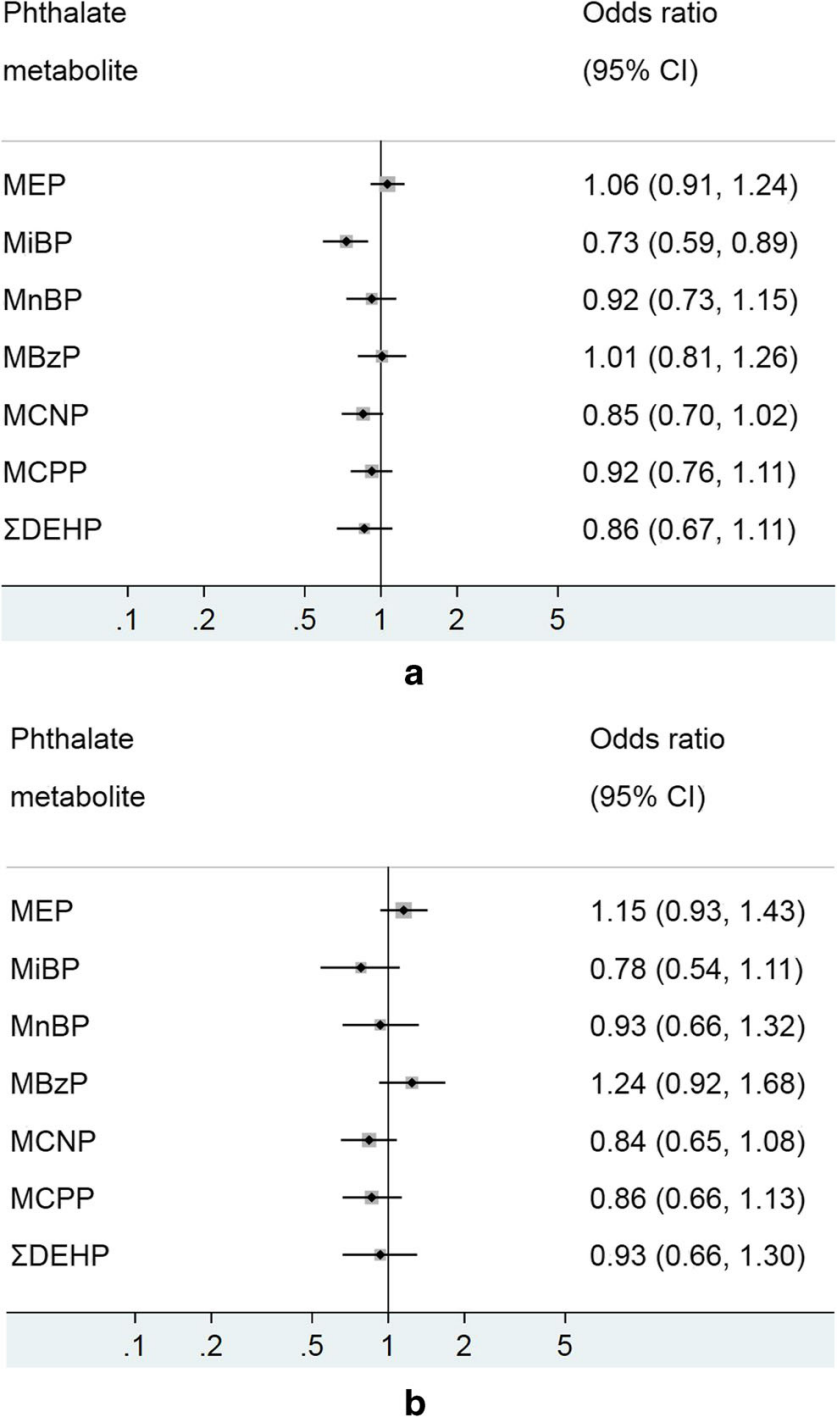
**a** Model 1 (crude)—associations of urinary phthalate metabolites with current asthma (spirometry measure) in adults, NHANES 2007–2012. Logistic regression modeling was used to access the effect of individual phthalate metabolites on asthma prevalence, with odds ratio (OR) presented for 1 log_10_ unit change in urinary phthalate concentration. **b** Model 2 (adjusted)—associations of urinary phthalate metabolites with current asthma (spirometry measure) in adults, NHANES 2007–2012. Logistic regression modeling was used to access the effect of individual phthalate metabolites on asthma prevalence, with odds ratio (OR) presented for 1 log_10_ unit change in urinary phthalate concentration. MEP mono-ethyl phthalate, MiBP mono-isobutyl phthalate, MnBP mono-n-butyl phthalate, MBzP mono-benzyl phthalate, MCNP mono-(carboxynonyl) phthalate, MCPP mono-(3-carboxylpropyl) phthalate, ΣDEHP molar sum of DEHP metabolites (MEHP, MEHHP, MECPP and MEOHP). All models were adjusted for age, sex, ethnicity/race, waist circumference, cotinine, poverty, and urinary creatinine

Association of urinary phthalate metabolites, and current asthma in adults were re-analysed using spirometry data, with the results presented in Fig. 5 a and b. MiBP was inversely associated with asthma in adults in the unadjusted model (0.73; 0.59–0.89), but the association did not reach statistical significance after adjusting for confounders. No other phthalate metabolites showed a significant relationship in either the crude nor the adjusted analyses.

**Fig. 6:**
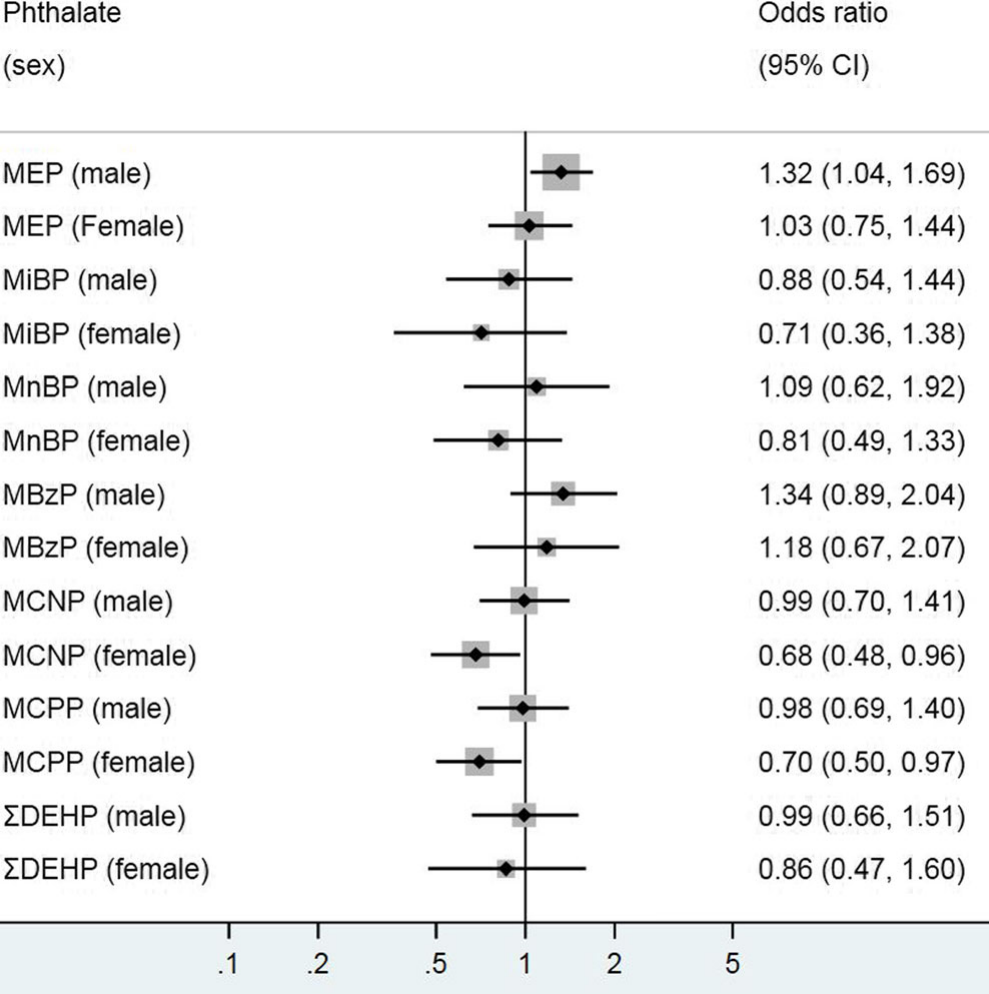
Associations between urinary phthalate metabolites and current asthma (spirometry measure in adults stratified by sex. Logistic regression modeling was used to access the effect of individual phthalate metabolites on asthma prevalence, with odds ratio (OR) presented for 1 log_10_ unit change in urinary phthalate concentration. MEP mono-ethyl phthalate, MiBP mono-isobutyl phthalate, MnBP mono-n-butyl phthalate, MBzP mono-benzyl phthalate, MCNP mono-(carboxynonyl) phthalate, MCPP mono-(3-carboxylpropyl) phthalate, ΣDEHP molar sum of DEHP metabolites (MEHP, MEHHP, MECPP and MEOHP). All models were adjusted for age, race/ethnicity, waist circumference, urinary creatinine and cotinine

The association of current asthma with MEP, MCPP and MCNP were not apparent in the overall model until after stratification by sex (Fig. 6). Similar to the result observed in children, a positive significant relationship was found between MEP and current asthma among adult males (1.32; 1.04–1.69) but not for females (1.03; 0.75–1.44). In contrast, MCPP and MCNP were negatively associated with current asthma in adult females alone.

Sensitivity analyses examining the exposure-response associations demonstrated increases or decreases in the odds of current asthma with increasing exposure category (Additional file 1: Tables S2, S3 and S4). Overall, the results were consistent with the primary analysis in both children and adults. There was a significant positive association between MBzP exposure and self-reported asthma in children for the highest tertile relative to the lowest tertile (1.99; 1.08–3.68), but not with any other phthalate metabolites. For male children, exposure to the highest tertile of MEP was significantly associated with over a 2-fold increased odds of self-reported asthma compared to the lowest tertile of MEP (2.38; 1.107–5.29). Similarly, adult males in the middle (1.64; 1.01–2.68) and highest tertiles (1.66; 1.07–2.2.59) had an elevated odds of asthma compared to those in the lowest tertiles. With the exception of MEP, no other phthalate metabolites showed a positive association with current asthma in males.

Results were, however, less consistent for adult females, with no significant relationship found using spirometry data as opposed to the inverse association found between MCNP and MCPP metabolites and current asthma in the primary analysis.

## Discussion

In this study, we observed no clear relationship between phthalate exposure and asthma, apart from one significant association between MBzP and self-reported asthma in children. Stratification by sex revealed that both boys and adult males are not at increased odds of current asthma following exposure to the majority of phthalate metabolites apart from the MEP metabolite. We found associations of MCNP and MCPP concentrations with reduced odds of asthma (defined using FEV_1_/FVC 70% cut off) among adult females. MiBP, MnBP and ΣDEHP were not significantly associated with either self-reported or objectively defined asthma (spirometry measure) in both children and adults.

Whyatt et al. (2014) examined the relationship between the diagnosis of asthma in children (aged 5–11 years, *n* = 300) and prenatal exposures BBzP, di-n-butyl phthalate (DnBP), DEHP and di-ethyl phthalate (DEP) using a longitudinal birth cohort of 727 women enrolled between 1998 and 2006. They found that maternal prenatal MBzP and MnBP concentrations—metabolites of HMW BBzP and DnBP, respectively—were significantly associated with the diagnosis of current asthma and with a history of asthma-like symptoms (Whyatt et al. 2014). The present study found a significant association between MBzP and self-reported asthma in children, but no relationship was found for MnBP.

A positive correlation was found between an HMW metabolite, DEHP exposure and asthma in settled dust (Gascon et al. 2015), but not with MBzP (Bornehag et al. 2004; Kolarik et al. 2008). A cross-sectional study of 623 Norwegian children aged 10 years old reported a significant relationship with the highest quartiles of MCNP and mono-(carboxyloctyl) phthalate (MCOP) (Bertelsen et al. 2013). However, we did not find a positive association of childhood asthma with DEHP or MCNP in either the crude or the adjusted analyses. This disparity may be attributed in part to the matrix examined, dust (Gascon et al. 2015; Bornehag et al. 2004; Kolarik et al. 2008) versus urine (Bertelsen et al. 2013). Another possible explanation might be the differences in specimen collection (i.e. the use of first-morning void (Bertelsen et al. 2013) as opposed to spot urine in the present study), as these may affect the concentrations of phthalate metabolites measured.

MBzP is a primary metabolite of BBzP, an HMW phthalate used in the manufacturing of toys, PVC materials, child care articles and personal care products, and for pharmaceutical coatings (Benjamin et al. 2017; Braun et al. 2013). While exposure to some phthalate compounds, particularly among asthmatics, may be via pharmaceuticals, Hoppin et al. (2013) suggested that the presence of MBzP in urine is unlikely to be as a result of the use of asthma medication since BBzP is not approved for pharmaceutical coatings. Compared with non-asthmatics, Hsu et al. (2012) (Hsu et al. 2012) demonstrated that asthmatic children had significantly higher levels of BBzP determined in settled dust, even after controlling for other indoor air pollutants. They proposed that the inhalation of BBzP may be an important pathway to the development or exacerbation of asthma in children in Taiwan (Hsu et al. 2012).

In a study using the NHANES data, Hoppin et al. (2013) showed that HMW phthalate metabolites, particularly MBzP, were positively associated with current asthma, current wheeze, current hay fever, and current rhinitis in adults (aged 18 years and older, *n* = 1596), but not in children (aged 6–17 years, *n* = 779). This disparity with our analysis may be attributed to the asthma definition and/or the measurement method used. Hoppin et al. (2013) separated the definition of current asthma and wheeze in their analysis, whereas the present study combined these self-reported measures. It is also possible that our findings for children may have occurred by chance alone since no other phthalate analysed showed a significant relationship in the adjusted models.

We did observe that the weighted geometric mean concentration of MBzP in children was two-fold the level seen in adults (Tables 4 and 5). This suggests that children are more exposed to MBzP than adults and may be at increased odds of asthma following this exposure. It is important to note that our findings for children should, however, be interpreted with caution. While one (MBzP metabolite) out of ten metabolites analysed was positively associated with asthma, this does not suggest any strong relationship between phthalates and asthma. For adults, using both self-reported and spirometry data of the present study, there were no associations between any phthalate metabolite and current asthma.

With respect to effect modification by sex, Ku et al. (2015) reported that exposure to MEP metabolite was significantly associated with an increased odds of asthma among boys, but not among girls; and this was reflected in our study. Analysis of 240 adult participants (140 females, 100 males; 20 to 60 years) of NHANES III revealed that MEP levels in urine were associated with a reduction in pulmonary function measures (FEV1, FVC) in adult males alone (Hoppin et al. 2004). A similar study of 3147 participants (aged between 6 and 49 years) found significant associations between MEP, MnBP, MCPP and ΣDEHP exposure, and a reduction in FEV_1_ or FVC in men (Cakmak et al. 2014). Although an inverse association was found between MCPP and MCNP metabolites and current asthma in adult females, our result for MEP was consistent with previous studies for both boys and adult males.

MEP is a primary metabolite of DEP, an LMW phthalate used in varieties of consumer products including fragrances and personal care products. Sex differences in MEP concentrations have been explained based on the use of these products, with evidence of higher MEP levels in females than in males (Saravanabhavan et al. 2014). However, our finding suggests that higher exposure to DEP may not explain the observed association among males. The sex-specific relationship may be attributed to either the endocrine disruptive ability of DEP in relation to sex differences in asthma prevalence (Buckley et al. 2018), or the hormonal influence of the chemical on the functioning of lungs and the immune systems. It is also plausible that the interactions between gene and environmental exposure to DEP may have resulted in sex-specific differences and the observed male susceptibility to asthma prevalence.

Although the pathways through which phthalates induce asthma in humans remain unclear (Whyatt et al. 2014), animal studies have provided stronger evidence of their deleterious effects. For example, the metabolites of HMW phthalates, especially MBzP and MEHP, were shown to bind with and activate the nuclear peroxisome proliferator-activated receptors (PPAR-alpha and PPAR-gamma), which play a significant role in certain physiological processes including airway remodeling and inflammation in rodents (Hurst and Waxman 2003). More recently, in vivo studies have demonstrated that DEHP induces Th2 and Th17 immune responses and airway inflammation in mice (Alfardan et al. 2018), and thymic stromal lymphopoietin (TSLP), Th2 immune response and interleukin-7 receptor in rats (Wang et al. 2018); all of which exacerbates asthma.

Some limitations of the study include the cross-sectional design of NHANES making it difficult to establish causality in the associations between phthalate exposures and asthma in children and adults. Phthalate measurements are prone to exposure misclassification via the use of a single spot urine sample per subject, which may not take into account the variation of within-person over time. Nevertheless, although phthalate metabolites have biological half-lives of less than a day (Jepsen et al. 2004; Ferguson et al. 2011), research has shown that, despite this temporal variability, the measurement of phthalate concentrations via a single spot urine sample may be a representative of long-term exposures (Teitelbaum et al. 2008). We were unable to repeat the analysis for children using spirometry data. This is because FEV_1_/FVC was considered a poor diagnostic test for childhood asthma due to a lack of accuracy and precision (Murray et al. 2016) and the limited number of children classified as asthmatics (< 2%) compared to non-asthmatics (> 98%) (Table 2). This prevented us from investigating if the observed association between MBzP metabolites and self-reported asthma in children were overestimated or due to chance. Finally, some children and adults with asthma, particularly of lower socio-economic status (SES), may not have received a diagnosis of asthma due to lack of healthcare; and thus, are unaware of their current asthma status.

However, the strengths of the study include using three NHANES waves based on a representative sample of the US population that is diverse in terms of geographical distribution, ethnic groups, age, and income. Current asthma was defined using both subjective (self-reported) and objective (spirometry) measures in adults. Both our primary and sensitivity analyses were robust in statistical modeling approaches and may be generalised to the US population.

## Conclusions

Urinary concentrations of phthalate metabolites were not significantly associated with current asthma in children and adults, apart from a single metabolite. Stratification by sex revealed that boys and adult males were at increased odds of asthma following exposure to only MEP; adult females were at decreased odds of asthma following exposure to MCNP and MCPP. Based on our findings, the potential adverse effect of phthalate exposure on asthma pathogenesis and/or exacerbations remains controversial, highlighting the need for a more comprehensive study on phthalate exposure and the occurrence of asthma; ideally, integrating a well-designed longitudinal follow-up analysis would be more informative.

## Electronic supplementary material

ESM 1 **Table S1**. Spearman’s rank correlation coefficients for all phthalate metabolite concentrations (*n* = 7523). **Table S2**. Sensitivity analysis estimating associations of tertiles between urinary phthalate metabolite and asthma (self-reported) in children. **Table S3**. Sensitivity analysis estimating associations of tertiles between urinary phthalate metabolite and asthma (self-reported) in adults. **Table S4**. Sensitivity analysis estimating associations of tertiles between urinary phthalate metabolite and asthma (spirometry measure) in adults. (DOCX 24 kb)

## Data Availability

The datasets used for this analysis are publicly available on the NHANES website: https://wwwn.cdc.gov/nchs/nhanes/search/datapage.aspx?Component=Demographics

## Abbreviations

ATS: American Thoracic Society
BBzP: benzylbutyl phthalate
BMI: body mass index
CDC: Centers for Disease Control and Prevention
CI: confidence interval
cm: centimetre
DALYs: disability-adjusted life years
DEHP: di-(2-ethylhexyl) phthalate
DEP: di-ethyl phthalate
ED: emergency department
ERS: European Respiratory Society
FEV_1_: forced expiratory volume in 1 s
FVC: forced vital capacity
HMW: high molecular weight
ISAAC: The International Study of Asthma and Allergies in Childhood
LMW: low molecular weight
LOD: limit of detection
LOD_max_: maximum limit of detection
MBzP: mono-benzyl phthalate
MCNP: mono-(carboxylnonyl) phthalate
MCOP: mono-(carboxyloctyl) phthalate
MCPP: mono-(3-carboxylpropyl) phthalate
MECPP: mono-(2-ethyl-5-oxohexyl) phthalate
MEHHP: mono-(2-ethyl-5-hydroxylhexyl) phthalate
MEHP: mono-(2-ethyl-5-hexyl) phthalate
MEOHP: mono-(2-ethyl-5-oxohexyl) phthalate
MEP: mono-ethyl phthalate
MiBP: mono-iso-butyl phthalate
MnBP: mono-n-butyl phthalate
NCHS: National Centre for Health Statistics
NHANES: National Health and Nutrition Examination Survey
NHS: National Health Service
OR: odds ratio
PIR: income to poverty ratio
PPARs: peroxisome proliferator-activated receptors
PVC: polyvinyl chloride
SES: socioeconomic status
TSLP: thymic stromal lymphopoietin
UK: United Kingdom
US: United States
YLLs: years of life lost
