# Supplementary material for: Phthalates and asthma in children and adults: US NHANES 2007–2012
Source: Environ Sci Pollut Res Int. 2019 Jul 31;26(27):28256–69. doi: 10.1007/s11356-019-06003-2 (PMC6791917; doi:10.1007/s11356-019-06003-2)
Supplement: Supplementary file 1 — Table S1. Spearman’s rank correlation coefficients for all phthalate metabolite concentrations (n = 7523). Table S2. Sensitivity analysis estimating associations of tertiles between urinary phthalate metabolite and asthma (self-reported) in children. Table S3. Sensitivity analysis estimating associations of tertiles between urinary phthalate metabolite and asthma (self-reported) in adults. Table S4. Sensitivity analysis estimating associations of tertiles between urinary phthalate metabolite and asthma (spirometry measure) in adults. (DOCX 24 kb) [file 11356_2019_6003_MOESM1_ESM.docx]

Phthalates and asthma in children and adults: US NHANES 2007-2012

Chinonso C. Odebeatu

Tim J. Taylor

Lora E. Fleming

Nicholas J. Osborne

Additional file 1

Table S1. Spearman’s rank correlation coefficients for all phthalate metabolite concentrations (n=7523)

|  | MEP | MiBP | MnBP | MBzP | MCNP | MCPP | MEHP | MEHHP | MEOHP | MECPP |
| --- | --- | --- | --- | --- | --- | --- | --- | --- | --- | --- |
| MEP | 1.00 |  |  |  |  |  |  |  |  |  |
| MiBP | 0.42 | 1.00 |  |  |  |  |  |  |  |  |
| MnBP | 0.44 | 0.78 | 1.00 |  |  |  |  |  |  |  |
| MBzP | 0.32 | 0.65 | 0.73 | 1.00 |  |  |  |  |  |  |
| MCNP | 0.25 | 0.42 | 0.411 | 0.39 | 1.00 |  |  |  |  |  |
| MCPP | 0.24 | 0.52 | 0.55 | 0.50 | 0.64 | 1.00 |  |  |  |  |
| MEHP | 0.29 | 0.51 | 0.52 | 0.44 | 0.39 | 0.47 | 1.00 |  |  |  |
| MEHHP | 0.36 | 0.60 | 0.65 | 0.57 | 0.46 | 0.57 | 0.78 | 1.00 |  |  |
| MEOHP | 0.36 | 0.63 | 0.67 | 0.59 | 0.47 | 0.59 | 0.77 | 0.98 | 1.00 |  |
| MECPP | 0.35 | 0.57 | 0.62 | 0.54 | 0.52 | 0.59 | 0.73 | 0.94 | 0.94 | 1.00 |

MEP: mono-ethyl phthalate; MiBP: mono-isobutyl phthalate; MnBP: mono-n-butyl phthalate; MBzP: mono-benzyl phthalate; MCNP: mono (carboxynonyl) phthalate; MCPP: mono (3-carboxylpropyl) phthalate; MEHP: mono (2-ethylhexyl) phthalate; MEHHP: mono (2-ethyl-5-hydroxylhexyl) phthalate; MEOHP: mono (2-ethyl-5-hydroxylhexyl) phthalate; MECPP: mono (2-ethyl-5-carboxypentyl) phthalate.

Table S2. Sensitivity analysis estimating associations between tertiles of urinary phthalate metabolites and asthma (self-reported) in children

| Children aged 6- <18 years | | | |
| --- | --- | --- | --- |
| OR^a^ (95% CI) | | | |
|  | Overall^b^ | Male^c^ | Female^c^ |
| ***MEP*** | | | |
| Lowest tertile | Ref | Ref | Ref |
| Middle tertile | 0.88 (0.56, 1.38) | 0.77 (0.38, 1.58) | 0.94 (0.47, 1.90) |
| Highest tertile | 1.35 (0.72, 2.52) | 2.38 (1.07, 5.29) | 0.83 (0.35, 1.94) |
| ***MiBP*** | | | |
| Lowest tertile | Ref | Ref | Ref |
| Middle tertile | 1.22 (0.64, 2.35) | 0.83 (0.36, 1.92) | 1.69 (0.46, 6.25) |
| Highest tertile | 1.34 (0.67, 2.66) | 1.18 (0.51, 2.72) | 1.49 (0.40, 5.59) |
| ***MnBP*** |  |  |  |
| Lowest tertile | Ref | Ref | Ref |
| Middle tertile | 1.50 (0.82, 2.76) | 1.26 (0.57, 2.75) | 1.64 (0.53, 5.09) |
| Highest tertile | 1.20 (0.66, 2.18) | 1.17 (0.51, 2.69) | 1.12 (0.40, 3.10) |
| ***MBzP*** |  |  |  |
| Lowest tertile | Ref | Ref | Ref |
| Middle tertile | 1.33 (0.62, 2.81) | 1.24 (0.39, 3.99) | 1.26 (0.48, 3.35) |
| Highest tertile | 1.99 (1.08, 3.68) | 1.84 (0.59, 5.69) | 1.59 (0.63, 4.01) |
| ***MCNP*** |  |  |  |
| Lowest tertile | Ref | Ref | Ref |
| Middle tertile | 1.33 (0.75, 2.35) | 1.24 (0.53, 2.94) | 1.27 (0.49, 3.28) |
| Highest tertile | 1.19 (0.64, 2.23) | 0.93 (0.31, 2.80) | 1.34 (0.62, 3.12) |
| ***MCPP*** |  |  |  |
| Lowest tertile | Ref | Ref | Ref |
| Middle tertile | 0.51 (0.25, 1.04) | 0.51 (0.21, 1.21) | 0. 43 (0.17, 1.14) |
| Highest tertile | 0.73 (0.36, 1.47) | 0.46 (0.19, 1.13) | 0.88 (0.35, 2.19) |
| ***ΣDEHP*** |  |  |  |
| Lowest tertile | Ref | Ref | Ref |
| Middle tertile | 0.86 (0.48, 1.57) | 0.86 (0.37, 2.05) | 0.98 (0.33, 2.43) |
| Highest tertile | 0.96 (0.52, 1.78) | 0.75 (0.37, 1.54) | 1.34 (0.56, 3.21) |

Table S3. Sensitivity analysis estimating associations between tertiles of urinary phthalate metabolite and asthma (self-reported) in adults

| Adults aged 18- <80 years | | | |
| --- | --- | --- | --- |
| OR^a^ (95% CI) | | | |
|  | Overall^b^ | Male^c^ | Female^c^ |
| ***MEP*** | | | |
| Lowest tertile | Ref | Ref | Ref |
| Middle tertile | 1.04 (0.69, 1.57) | 0.80 (0.40, 1.57) | 1.18 (0.69, 2.03) |
| Highest tertile | 1.12 (0.71, 1.78) | 1.06 (0.53, 2.14) | 1.18 (0.68, 2.06) |
| ***MiBP*** | | | |
| Lowest tertile | Ref | Ref | Ref |
| Middle tertile | 0.83 (0.54, 1.27) | 1.48 (0.82, 2.68) | 0.62 (0.37, 1.05) |
| Highest tertile | 0.81 (0.50, 1.28) | 1.42 (0.77, 2.59) | 0.65 (0.39, 1.07) |
| ***MnBP*** |  |  |  |
| Lowest tertile | Ref | Ref | Ref |
| Middle tertile | 0.84 (0.59, 1.19) | 0.76 (0.43, 1.34) | 0.89 (0.57, 1.40) |
| Highest tertile | 0.90 (0.58, 1.41) | 0.98 (0.48, 1.98) | 0.91 (0.54, 1.50) |
| ***MBzP*** |  |  |  |
| Lowest tertile | Ref | Ref | Ref |
| Middle tertile | 0.86 (0.55, 1.32) | 1.21 (0.69, 2.13) | 0.71 (0.41, 1.23) |
| Highest tertile | 0.93 (0.64, 1.36) | 1.03 (0.47, 2.27) | 0.88 (0.55, 1.41) |
| ***MCNP*** |  |  |  |
| Lowest tertile | Ref | Ref | Ref |
| Middle tertile | 1.19 (0.77, 1.76) | 0.98 (0.50, 1.94) | 1.29 (0.79, 2.10) |
| Highest tertile | 1.27 (0.93, 1.73) | 1.28 (0.74, 2.21) | 1.27 (0.79, 2.00) |
| ***MCPP*** |  |  |  |
| Lowest tertile | Ref | Ref | Ref |
| Middle tertile | 1.15 (0.74, 1.77) | 1.37 (0.64, 2.99) | 1.04 (0.61, 1.78) |
| Highest tertile | 1.29 (0.82, 2.02) | 0.84 (0.44, 1.61) | 1.50 (0.87, 2.57) |
| ***ΣDEHP*** |  |  |  |
| Lowest tertile | Ref | Ref | Ref |
| Middle tertile | 1.07 (0.73, 1.55) | 0.83 (0.40, 1.72) | 1.22 (0.75, 2.00) |
| Highest tertile | 1.05 (0.67, 1.64) | 0.84 (0.36, 1.99) | 1.18 (0.71, 1.98) |

Table S4. Sensitivity analysis estimating associations between tertiles of urinary phthalate metabolites and asthma (spirometry measure) in adults

| Adults aged 18- <80 years | | | |
| --- | --- | --- | --- |
| OR^a^ (95% CI) | | | |
|  | Overall^b^ | Male^c^ | Female^c^ |
| ***MEP*** | | | |
| Lowest tertile | Ref | Ref | Ref |
| Middle tertile | 1.34 (1.01, 1.80) | 1.64 (1.01, 2.68) | 1.02 (0.61, 1.71) |
| Highest tertile | 1.30 (0.90, 1.86) | 1.66 (1.07, 2.59) | 0.91 (0.51, 1.62) |
| ***MiBP*** | | | |
| Lowest tertile | Ref | Ref | Ref |
| Middle tertile | 0.98 (0.75, 1.30) | 0.90 (0.64, 1.27) | 1.12 (0.69, 1.80) |
| Highest tertile | 0.80 (0.56, 1.13) | 0.99 (0.57, 1.71) | 0.66 (0.37, 1.17) |
| ***MnBP*** |  |  |  |
| Lowest tertile | Ref | Ref | Ref |
| Middle tertile | 1.14 (0.91, 1.43) | 0.92 (0.65, 1.30) | 1.57 (0.99, 2.48) |
| Highest tertile | 0.79 (0.57, 1.11) | 0.90 (0.51, 1.59) | 0.80 (0.46, 1.39) |
| ***MBzP*** |  |  |  |
| Lowest tertile | Ref | Ref | Ref |
| Middle tertile | 1.12 (0.84, 1.48) | 1.29 (0.85, 1.97) | 0.89 (0.53, 1.51) |
| Highest tertile | 1.06 (0.75, 1.51) | 1.08 (0.65, 2.79) | 1.05 (0.60, 1.84) |
| ***MCNP*** |  |  |  |
| Lowest tertile | Ref | Ref | Ref |
| Middle tertile | 1.12 (0.83, 1.52) | 1.00 (0.68, 1.48) | 1.32 (0.80, 2.16) |
| Highest tertile | 0.91 (0.66, 1.25) | 1.05 (0.70, 1.57) | 0.74 (0.47, 1.21) |
| ***MCPP*** |  |  |  |
| Lowest tertile | Ref | Ref | Ref |
| Middle tertile | 1.02 (0.73, 1.43) | 1.15 (0.81, 1.63) | 0.89 (0.52, 1.54) |
| Highest tertile | 1.02 (0.74, 1.41) | 1.14 (0.77, 1.69) | 0.88 (0.58, 1.33) |
| ***ΣDEHP*** |  |  |  |
| Lowest tertile | Ref | Ref | Ref |
| Middle tertile | 1.10 (0.78, 1.56) | 1.26 (0.80, 1.97) | 0.88 (0.51, 1.55) |
| Highest tertile | 0.82 (0.61, 1.11) | 0.92 (0.56, 1.50) | 0.70 (0.42, 1.18) |

OR: odds ratio; CI: confidence interval;

^a^ OR: odds ratio compared to the lowest tertiles of creatinine-corrected phthalate concentrations.

^b^ Models are adjusted for age, sex, ethnicity/race, waist circumference, poverty, cotinine

^c^ Models are adjusted for age, ethnicity/race, waist circumference, poverty, cotinine.
